# Disentangling direct and weight-mediated effects of physical activity on health-related quality of life in a 12-month real-world digital lifestyle intervention

**DOI:** 10.1007/s11136-026-04425-3

**Published:** 2026-10-22

**Authors:** Isabel McCann, Anu Joki, Laura-Unnukka Suojanen, Mikko S. Venäläinen, Kirsi H. Pietiläinen, Aila J. Ahola

**Affiliations:** 1 University of South Florida Morsani College of Medicine, Tampa, FL USA; 2 Obesity Research Unit, Research Program for Clinical and Molecular Metabolism, Faculty of Medicine, University of Helsinki, Biomedicum 1, Haartmaninkatu 8, PO Box 63, 00014 Helsinki, Finland; 3 Healthy Weight Hub, Abdominal Centre, Helsinki University Hospital and University of Helsinki, Helsinki, Finland; 4 Department of Medical Physics, Turku University Hospital and University of Turku, Turku, Finland

**Keywords:** Digital lifestyle intervention, Health-related quality of life, Obesity, Physical activity

## Abstract

**Purpose:**

To examine whether participation in a 12-month digital lifestyle program was associated with increased physical activity and whether physical activity was associated with health-related quality of life (HRQoL) independently of weight loss.

**Methods:**

Self-reported weight and height were used to calculate body mass index (BMI). Physical activity (metabolic equivalent of task hours, MET-h/day) was assessed using a web-based questionnaire at baseline, 3, 6, 9, and 12 months. HRQoL was measured with the RAND-36 at baseline, 6, and 12 months yielding eight domains and physical and mental component scores. Longitudinal mixed-effects models and mediation analyses were used to examine associations between physical activity, BMI, and HRQoL over time.

**Results:**

In 1732 individuals (82.9% women, median age 52 years, BMI 39.0 kg/m^2^), estimated marginal mean physical activity increased from 2.16 to 3.06 MET-h/day (*p* < 0.001). Periods of higher-than-usual physical activity were associated with lower BMI within individuals (B = − 0.032, 95% CI − 0.053 to − 0.012). Higher physical activity was also associated with better scores in most HRQoL dimensions, including physical and mental component scores. Indirect associations via BMI were largely limited to physical domains, whereas direct associations predominated: 84.9% of the total association with physical functioning and 88.0% of that with bodily pain were attributable to the direct pathway.

**Conclusion:**

Participation in a digital lifestyle program was associated with increased physical activity, and physical activity was associated with better HRQoL largely independently of weight change, although follow-up was incomplete and the magnitude of the individual associations was modest. Physical activity merits attention as a treatment goal alongside weight management.

**Trial registration:**

The trial is registered at clinicaltrials.gov (Clinical Trials Identifier NCT04019249). Registration date 12 Jul 2019. Retrospectively registered.

**Supplementary Information:**

The online version contains supplementary material available at https://doi.org/10.1007/s11136-026-04425-3.

## Introduction

Obesity is associated with increased morbidity and mortality and with lower health-related quality of life (HRQoL) [1–3]. HRQoL reflects the perceived impact of health status on physical, mental, and social well-being. Individuals with obesity report, on average, lower physical functioning, vitality, and psychological well-being [4]. Contemporary obesity management therefore increasingly emphasizes broader health and well-being alongside weight-related outcomes, with HRQoL recognized as an important therapeutic target [5].

Lifestyle modification remains the cornerstone of obesity care, with increasing physical activity representing a key behavioural target influencing both physiological health and perceived well-being [6]. Physical activity may improve HRQoL through mechanisms partly independent of weight loss, such as enhanced functional capacity, mood improvements, and reduced symptom burden [7]. Weight loss may also contribute to improvements in HRQoL through reductions in mechanical burden, improved physical functioning, and changes in metabolic health, with effects typically more evident in physical than mental domains. However, the extent to which HRQoL improvements stem from increased physical activity versus weight loss remains unclear.

In recent years, digital health interventions have emerged as scalable tools to support lifestyle change. Compared to face-to-face programs, they reduce logistical barriers and provide support to large populations at lower costs [8, 9]. For weight loss, digital programs are comparable to face-to-face interventions [10]. However, evidence on their effectiveness in promoting physical activity is mixed [11, 12]. Less is known about how changes in physical activity in these real-world digital programs translate into HRQoL benefits, and whether improvements are driven primarily by physical activity or weight loss. This distinction is clinically relevant, as it may inform how interventions are structured and which behavioural components should be prioritized.

Using data from the 12-month digital Healthy Weight Coaching (HWC) program, we recently demonstrated that participation in the program was associated with improvements across all HRQoL domains [13]. Building on these observations, a simple conceptual framework guided the present study: physical activity was assumed to influence HRQoL through a direct pathway, reflecting functional capacity, symptom burden, and psychological well-being, and an indirect pathway operating through weight loss. Physical activity was accordingly treated as the primary exposure, body mass index (BMI) as a potential mediator, and HRQoL as the outcome, guiding the use of longitudinal mediation analysis. We investigated whether participation in the program was associated with increased physical activity and whether such increases were linked to lower BMI. Our primary objective was to assess whether HRQoL changes were driven mainly by physical activity or weight loss. We hypothesized that physical activity would be associated with improvements in both the physical and mental HRQoL components, whereas weight loss would primarily mediate improvements in the physical domains.

## Methods

### The program

The HWC, a 12-month digital lifestyle intervention based on behavioural therapy principles, is delivered through the HealthyWeightHub.fi platform [14]. The program consists of 52 automated weekly sessions covering modules on diet, physical activity, sleep, and psychological well-being. To complement the automated content, each participant is assigned a trained health care professional who provides standardized yet individually responsive online coaching throughout the program. Physical activity is addressed in multiple weekly modules covering increases in daily activity, reduction of sedentary time, and education on how physical activity relates to sleep and recovery, pain management, emotional wellbeing, and exercising with chronic conditions. Participants are encouraged to reflect on the role of physical activity in lifestyle change, supported in selecting activity levels suitable to their functional capacity, and provided with resources including exercise videos, movement reminders, and a voluntary activity log. A more detailed description of the program has been published elsewhere [15, 16]. The Ethics Committee of the Helsinki and Uusimaa Hospital District approved the study protocol (327/13/03/00/2015, GDPR update 587/2019). Informed consent was obtained prior to participation.

### Population

Enrolment in the HWC program requires referral by any physician licensed in Finland. Eligible participants were ≥ 18 years of age, with BMI ≥ 25 kg/m^2^, internet access, and willingness to join an online lifestyle program. Individuals for whom the program might be unsafe (e.g., cognitive impairments, cancer, pregnancy) were excluded, as treatment may be better managed in clinics. Data were available for consenting individuals enrolled from October 2016 to September 2020 who completed a 2-week trial. As this was a real-world clinical program, data were available only for participants who provided informed consent for research use, and information on individuals not consenting or not proceeding to the program was not accessible. We excluded from the current analyses those using other weight loss methods (gastric bypass surgery, gastric balloon, obesity management medication, or very low-calorie diet) and those with missing physical activity data at baseline. Age and sex were sourced from the Finnish national register.

### Measurements

#### Weight and height

Participants self-reported weight at baseline and, thereafter, could report it daily, with weekly reporting encouraged. Weight data were interpolated at 3, 6, 9, and 12 months. Height was self-reported at baseline, and BMI was calculated as kg/m^2^ at each time point. For each follow-up timepoint, we calculated percentage weight change from baseline.

#### Physical activity

Participants reported intensity (“how strenuous is your physical activity?”), single-session duration (“how long is a typical session of your physical activity?”), and frequency (“how often do you engage in physical activity?”) of leisure-time physical activity using a brief self-reported questionnaire, adapted from a previously published paper [17], at baseline and at 3, 6, 9, and 12 months. Intensity was reported at three levels; low (e.g., walking, 4 metabolic equivalents (MET)), medium (e.g., bicycling, 8 MET), and high (i.e., exercising “to exhaustion,” 13 MET). Single-session duration was reported on a four-level scale; <15, 15–30, 31–60, and > 60 min and converted respectively to 0.12, 0.375, 0.75, and 1.5 h. Frequency was reported on a seven-level scale; never, < 1/month, 1–3/month,1/week, 2–3/week, 4–5/week, and almost every day. These were converted to 0, 11, 24, 52,130, 234, and 365 days/year, respectively. Total daily physical activity was calculated in MET-hours per day (MET-h/day) [(intensity (MET) ⋅ single-session duration (hours) ⋅ frequency (days/year))/365]. MET-h/day was calculated at baseline and at 3, 6, 9, and 12 months.

#### Health-related quality of life

Participants completed the validated Finnish RAND-36 at baseline as well as at 6- and 12-month follow-up points [18]. The RAND-36 is a generic HRQoL instrument comparable to the SF-36, consisting of 36 items assessing perceived health and functioning over the preceding four weeks. Items cover multiple dimensions of physical and mental health and are phrased as questions regarding limitations in daily activities, pain, emotional and physical well-being, and general health perceptions. In accordance with established scoring procedures in which each domain score is the mean of the answered items within a permitted number of missing items, item responses were transformed into eight HRQoL domains: physical functioning, role limitations due to physical health, bodily pain, general health, vitality, social functioning, role limitations due to emotional problems, and mental health. Scores for each domain range from 0 to 100, with higher values reflecting more favourable self-rated health. Using the approach by Ware et al. [19], we computed the physical and mental component summary scores from the eight HRQoL domains to represent the core dimensions of HRQoL. All ten resulting variables were retained as continuous measures for the analyses.

### Statistical analyses

Basic characteristics are reported as frequencies and percentages for categorical variables and medians (interquartile ranges) for continuous variables. Between-group comparisons were conducted using the chi-squared test for categorical variables and the Mann-Whitney U-test for continuous variables. These comparisons were made between participants included in and excluded from the analyses, and between those with and without follow-up HRQoL data at 6 and 12 months. To assess the statistical significance of changes in physical activity over time, a linear mixed model was used, with time included as a fixed effect and participant ID as a random effect, to account for repeated measurements within individuals. Longitudinal associations between physical activity and BMI were analysed using linear mixed models estimated using restricted maximum likelihood. Repeated observations obtained at baseline and at 3-, 6-, 9-, and 12-month follow-up were modelled within participants using a first-order autoregressive covariance structure (AR(1)). Physical activity variables were decomposed into between-person (participant-specific mean values across follow-up) and within-person (deviations from participant-specific mean values) components to distinguish associations attributable to differences between individuals from associations attributable to changes within individuals over time. All four physical activity variables were treated as continuous, and each was analysed in a separate model. In an additional model, the three components of physical activity were analysed simultaneously to assess their independent contributions; total physical activity was not included in the model, as it is the product of the three components. These analyses were conducted using IBM SPSS Statistics for Windows, version 31.0.2.0 (IBM Corp., Armonk, NY, USA).

Longitudinal mediation analyses were conducted using linear mixed-effects modelling in R (packages lme4 and lmerTest) to examine whether the association between physical activity and HRQoL over time was mediated by BMI. Mediation was assessed within a multilevel (within-person) framework by fitting separate mixed-effects models for each pathway: (i) the association between within-person changes in physical activity and BMI (a-path), (ii) the association between BMI and HRQoL adjusting for physical activity (b- and c’-paths), and (iii) the total association between physical activity and HRQoL (c-path). Physical activity, BMI, HRQoL variables were decomposed into participant-specific mean (between-person effects) and deviations from these means (within-person effects), and both components were entered simultaneously into the models. The inclusion of both within-person and between-person components ensured that mediation effects reflected intra-individual changes over time rather than differences between individuals. Indirect effects (a × b-path) were estimated using Monte Carlo simulation based on model parameter estimates (5,000 simulations), and 95% confidence intervals were derived from the empirical distribution of simulated effects. Two-sided p-values for the indirect effects were calculated based on the simulated distribution. All longitudinal analyses were adjusted for age, sex, and time (included as fixed effects).

All available observations were included in all longitudinal analyses, as permitted by the mixed-effects modelling framework, which assumes data to be missing at random conditional on the observed data; no imputation was performed. Model assumptions were evaluated by inspecting residual distributions and plots to assess normality and homoscedasticity. The level of statistical significance was set at *p* < 0.05. The primary outcome was change in HRQoL, in line with the primary objective of assessing whether these changes were driven by physical activity or weight loss. Secondary outcomes were considered exploratory; therefore, no adjustments for multiple comparisons were applied. The figure was created using Inkscape (version 1.4.3; Inkscape Project).

## Results

### Population

Altogether, 2157 individuals were enrolled to the program. Of these, we excluded 182 individuals who used additional weight loss methods, and 243 who didn’t have baseline physical activity data (Supplementary Fig. 1). Of those included 1732 (80.3%), 82.9% were women (Table 1). The median age was 52 years, and the median BMI was 39.0 kg/m^2^ with most (98.2%) having obesity (BMI ≥ 30 kg/m^2^). Compared to individuals included in the analyses, those excluded were heavier (110 kg vs. 114 kg, *p* = 0.012; 39.0 kg/m^2^ vs. 40.1 kg/m^2^, *p* < 0.001) but had comparable sex distribution and median age (Supplementary Table 1). All 1732 participants had valid baseline data in at least one HRQoL domain (*n* = 1674–1718 across the eight domains and 1542 for both component scores). Numbers of observations at each time point are shown in Supplementary Table 2.

**Table 1:** Baseline characteristics of the study participants

	*N* = 1732		
	*N* of observations	*N* (%) or median (interquartile range)	Range
Women	1732	1436 (82.9)	
Age, years	1732	52 (42–59)	21–81
Weight, kg	1732	110 (98–126)	60–284
Body mass index, kg/m^2^	1732	39.0 (35.3–43.5)	26.5–78.7
Physical activity, MET-h/day	1732	1.5 (0.4–3.0)	0–12.5
Health-related quality life domains			
Physical functioning	1718	75 (55–85)	0–100
Role limitations due to physical health	1710	50 (25–100)	0–100
Bodily pain	1703	58 (35–78)	0–100
General health	1715	45 (35–60)	0–100
Vitality	1712	55 (40–65)	0–100
Social functioning	1675	75 (63–100)	0–100
Role limitations due to emotional problems	1674	100 (33–100)	0–100
Mental health	1712	68 (56–80)	8–100
Physical component score	1542	41 (35–47)	11–68
Mental component score	1542	50 (39–57)	8–73

MET-h/day, metabolic equivalent of task hours per day

Compared with those without follow-up HRQoL data, participants with HRQoL data at 6 and 12 months were more often women, older, and had lower baseline weight and BMI and higher baseline physical activity (Supplementary Table 3). Baseline physical functioning, role limitations due to physical health, and the physical component score were similar between the groups, whereas general health, vitality, mental health, and the mental component score were slightly higher among those with follow-up data.

### Physical activity across the program

Total physical activity levels increased, with estimated marginal means (95% CI) from the linear mixed model of 2.16 (2.04–2.27), 2.71 (2.56–2.85), 2.65 (2.48–2.82), 2.78 (2.57–2.99), 3.06 (2.80–3.32) MET-h/day at baseline and at the 3-, 6-, 9-, and 12-month assessments, respectively. The linear mixed model showed that this increase was statistically significant (F[4, 554.9] = 26.6, *p* < 0.001).

### Associations between physical activity and BMI

Higher average physical activity levels were associated with lower BMI (B = − 0.652 [95% CI − 0.779 to − 0.526], *p* < 0.001) across participants (Table 2). Within individuals, lower BMI was observed during periods of higher-than-usual physical activity (B = − 0.032 [95% CI − 0.053 to − 0.012], *p* = 0.002). Among individual components of physical activity, greater frequency, duration, and intensity were all associated with lower BMI. However, only for frequency was this within-person pattern observed, with lower BMI during periods when a participant’s activity frequency was higher than usual. Full models are shown in Supplementary Table 4.

**Table 2:** Longitudinal associations between physical activity and body mass index across the 12-month program

	Between-person		Within-person	
	B (95% confidence interval)	*p*	B (95% confidence interval)	*p*
Metabolic equivalent of task hours/day	− 0.652 (− 0.779 to − 0.526)	< 0.001	− 0.032 (− 0.053 to − 0.012)	0.002
Physical activity—frequency	− 0.012 (− 0.015 to − 0.009)	< 0.001	− 0.001 (− 0.001 to − 0.001)	0.009
Physical activity—single-session duration	− 5.216 (− 6.068 to − 4.365)	< 0.001	− 0.084 (− 0.206–0.038)	0.178
Physical activity—intensity	− 0.338 (− 0.520 to − 0.156)	< 0.001	0.017 (− 0.006–0.040)	0.154

Linear mixed model was used to examine longitudinal associations between physical activity (independent variables) and body mass index (outcome) across five time points (baseline, 3, 6, 9, and 12 months). Adjusted for time, age and sex. Each physical activity variable was analysed in a separate model. Between-person estimates reflect whether individuals with higher average physical activity levels tend to have lower body mass index, whereas within-person estimates reflect whether changes in physical activity within an individual are associated with changes in body mass index over time

When the three components were analysed simultaneously, higher average frequency and single-session duration remained independently associated with lower BMI, whereas the association with intensity was no longer evident (Supplementary Table 5). Within individuals, frequency remained the only component associated with BMI over time.

### Longitudinal association between physical activity and HRQoL, mediation via weight loss

**Fig. 1:**
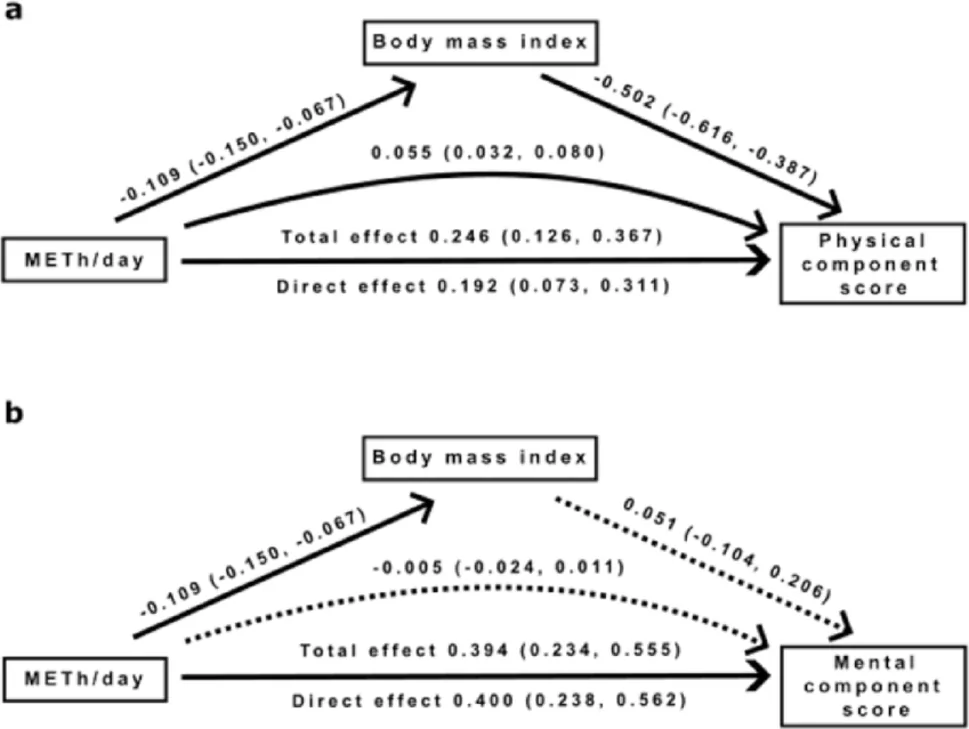
Within-person longitudinal associations between total daily physical activity (MET-h/d) and **a** physical component score and **b** mental component score, with body mass index as a mediator. Estimates are based on linear mixed-effects models in which variables were decomposed into within- and between-person components; the figure displays within-person associations. Models were adjusted for age, sex, and time (baseline, 6, and 12 months). Solid lines indicate statistically significant associations and dashed lines non-significant associations

Periods of higher-than-usual physical activity were consistently associated with lower BMI (Table 3, a-paths). In turn, lower BMI was associated with favourable changes in several HRQoL domains, including physical functioning, bodily pain, general health, vitality, social functioning, and physical component score (b-paths). Consequently, significant indirect effects of physical activity through BMI were observed for these same outcomes (a × b-paths). The total effects of physical activity were significant for nearly all HRQoL domains (physical functioning, bodily pain, general health, vitality, role limitations due to emotional problems, mental health, and both physical and mental component scores) (c-paths). Significant direct effects of physical activity were observed for these same eight HRQoL outcomes (c'-paths), indicating that increases in physical activity were associated with broad improvements in HRQoL independent of weight loss. The paths for the models with physical and mental component scores as outcomes are presented in Fig. 1a and b, respectively.

**Table 3:** Longitudinal mediation effects of physical activity on HRQoL via body mass index over the program

HRQoL	*N*	a-path	b-path	a × b-path	c-path	c′-path	PA-attrib. eff.
		B (95% CI)	B (95% CI)	B (95% CI), *p*	B (95% CI), *p*	B (95% CI), *p*	%
PF	1721	− 0.106 (− 0.145, − 0.066)	− 1.372 (− 1.591, − 1.153)	0.146 (0.088, 0.206), < 0.001	0.959 (0.726, 1.191), < 0.001	0.814 (0.586, 1.041), < 0.001	84.9
RP	1716	− 0.107 (− 0.147, − 0.067)	− 0.382 (− 1.020, 0.259)	0.042 (− 0.028, 0.116), 0.241	− 0.089 (− 0.753, 0.575), 0.793	− 0.130 (− 0.797, 0.538), 0.703	NA
BP	1715	− 0.107 (− 0.147, − 0.068)	− 0.713 (− 1.070, − 0.360)	0.077 (0.034, 0.127), < 0.001	0.638 (0.275, 1.000), < 0.001	0.561 (0.198, 0.925), 0.003	88.0
GH	1719	− 0.106 (− 0.145, − 0.067)	− 1.020 (− 1.240, − 0.804)	0.109 (0.064, 0.157), < 0.001	1.210 (0.979, 1.440), < 0.001	1.100 (0.873, 1.330), < 0.001	91.0
VT	1719	− 0.105 (− 0.144, − 0.066)	− 0.623 (− 0.877, − 0.369)	0.066 (0.033, 0.104), < 0.001	1.050 (0.793, 1.320), < 0.001	0.989 (0.728, 1.250), < 0.001	93.8
SF	1679	− 0.102 (− 0.142, − 0.062)	− 0.374 (− 0.731, − 0.017)	0.039 (0.002, 0.081), < 0.05	0.266 (− 0.104, 0.635), 0.159	0.228 (− 0.143, 0.598), 0.229	NA
RE	1680	− 0.100 (− 0.140, − 0.060)	0.191 (− 0.381, 0.763)	− 0.019 (− 0.083, 0.037), 0.529	1.160 (0.571, 1.750), < 0.001	1.180 (0.587, 1.780), < 0.001	101.6
MH	1719	− 0.105 (− 0.144, − 0.066)	− 0.222 (− 0.448, 0.004)	0.024 (− 0.001, 0.050), 0.056	0.691 (0.458, 0.923), < 0.001	0.667 (0.434, 0.901), < 0.001	96.6
PCS	1566	− 0.109 (− 0.150, − 0.067)	− 0.502 (− 0.616, − 0.387)	0.055 (0.032, 0.080), < 0.001	0.246 (0.126, 0.367), < 0.001	0.192 (0.073, 0.311), 0.002	77.8
MCS	1566	− 0.109 (− 0.150, − 0.067)	0.051 (− 0.104, 0.206)	− 0.005 (− 0.024, 0.011), 0.536	0.394 (0.234, 0.555), < 0.001	0.400 (0.238, 0.562), < 0.001	101.4

Analyses were conducted using longitudinal mixed-effects mediation models in R (lme4 and lmerTest packages). Physical activity (metabolic equivalent of task hours/ day), body mass index (BMI), and Health-Related Quality of Life (HRQoL) variables were decomposed into participant-specific means (between-person effects) and deviations from these means (within-person effects). Mediation effects thus represent intra-individuals changes over time. Separate mixed-effects models were fitted for the a-path (physical activity → BMI), b- and c’-paths (BMI and physical activity → HRQoL), and c-path (physical activity → HRQoL). Participant-specific random intercepts accounted for repeated observations. Indirect effects (a × b) were estimated using Monte Carlo simulation based on 5000 draws from the parameter distributions. Models were adjusted for time, age, and sex. N, the number of participants included in each mediation analysis; a-path, association between within-person physical activity and BMI; b-path, association between within-person BMI and HRQoL; c′-path, direct effect of physical activity on HRQoL controlling for BMI; a × b, indirect effect via BMI; c-path, total effect of physical activity on HRQoL; PA-attrib. eff., total effect attributable to physical activity (calculated for outcomes with a statistically significant total effect, c-path); B, unstandardized beta; CI, confidence interval; PF, physical functioning; RP, role limitations due to physical health; BP, bodily pain; GH, general health; VT, vitality; SF, social functioning; RE, role limitations due to emotional problems; MH, mental health; PCS, physical component score; MCS, mental component score

Across models, the contribution of BMI-mediated effects was modest compared with the direct effects of physical activity. For example, 84.9% of the total association with physical functioning and 88.0% of the association with bodily pain were attributable to the direct pathway. For role limitations due to emotional problems and mental component score, the indirect pathway through BMI operated in an opposite direction, such that reductions in BMI were associated with small, non-significant decreases in these scores. Consequently, the direct pathway accounted for more than 100% of the total association, as concurrent weight loss acted in the opposite direction.

## Discussion

In this sample of individuals with overweight or obesity, participation in a 12-month real-world digital lifestyle intervention was associated with an increase in physical activity, and periods of higher-than-usual physical activity were associated with lower BMI. We also observed that within-person increases in physical activity were independently associated with improvements in nearly all HRQoL domains, including both physical and mental component scores. Importantly, these associations were driven predominantly by direct effects of physical activity, whereas weight loss played a secondary mediating role that was mainly confined to physical HRQoL domains.

Reductions in BMI mediated the associations between physical activity and physical functioning, bodily pain, general health, vitality, social functioning, and the physical component score. However, the mediated share of the total association was small, 15% for physical functioning and 12% for bodily pain, and no mediation was evident for the mental component score. This pattern sits within a literature that has reached mixed conclusions [20–22]. Weight loss has been associated with HRQoL gains, with the largest effects among those with the highest baseline BMI [20]. However, a larger meta-analysis did not consistently support such an association [21], and in non-surgical interventions achieving ≥5% weight loss, benefits appear largely confined to physical HRQoL domains, with the causal nature of this association remaining uncertain [22]. In light of these mixed findings, some behavioural weight management trials have examined whether HRQoL improvements can be attributed to weight loss per se, with results suggesting that participants often experience broad and sustained HRQoL improvements that are not strongly dependent on the degree of weight loss [23, 24].

In the present study, increases in physical activity were directly associated with eight of the ten HRQoL outcomes, independently of concurrent weight loss. This is supported by, and extends, the existing trial evidence. In a randomized controlled trial of 73 individuals with obesity, both moderate-intensity continuous training and combining this training with high-intensity interval training produced comparable improvements in general health and several other HRQoL domains, indicating that structured aerobic exercise per se, rather than exercise intensity, may be sufficient to elicit meaningful improvements in perceived health and obesity-related quality of life [25]. Consistent with this, in a trial in which 6–7% weight loss was achieved through either diet or exercise, larger gains in self-perceived health were seen when the weight loss was achieved through exercise [26]. Not all evidence points in the same direction: among postmenopausal women with overweight or obesity, a combined diet and exercise program improved more HRQoL domains over 12 months than diet or exercise alone, and the overall HRQoL gains were most strongly associated with the magnitude of weight loss [27]. Importantly, these trials were small and short and were designed to compare interventions rather than to separate the contributions of activity and weight change. The present analysis complements them by examining within-person variation in more than 1500 individuals receiving routine care, and by estimating the direct and weight-mediated pathways simultaneously.

Weight loss primarily mediated associations with physical HRQoL domains, while mediation effects for mental domains were generally absent, indicating that improvements in mental HRQoL were largely independent of weight loss. This partly echoes prior reports that weight reduction does not uniformly translate into better mental HRQoL [21, 22] or may even relate to declines in specific contexts [28, 29]. For example, Müller-Nordhorn et al. [28], observed that weight loss was associated with a decline in mental HRQoL, particularly among individuals with normal weight, and Fine et al. [29] similarly reported a decrease in mental HRQoL among elderly women who lost 2–9 kg. However, important differences distinguish our findings from these two studies: unlike prior work where unintentional weight loss, potentially reflecting underlying illness or depressive symptoms, was suggested as a plausible driver of declining mental HRQoL, participants in the current study engaged in an intervention with the explicit goal of weight reduction. Notably, among individuals with overweight or obesity, Fine et al. [29] also observed that weight gain was associated with worsening mental HRQoL, suggesting that the relationship between body weight change and mental HRQoL may not be linear or causally straightforward. Overall, even in the context of intentional weight loss, as seen in the current study, reductions in body weight may not necessarily confer mental HRQoL benefits.

Participation in the HWC program was associated with an increase in physical activity, one of the core pillars of the intervention alongside diet, mental health, and rest/recovery [15]. On average, participants increased their physical activity by approximately 0.9 MET-hours per day, which corresponds to roughly 15 min of additional moderate-intensity activity such as brisk walking. Applying the model estimates to an increase of this size gives a BMI lower by about 0.03 units and a gain of roughly one point on the 0-100 HRQoL domain scales. For the physical and mental component scores the corresponding gains were 0.2–0.4 points. Only a small part of these gains operated through BMI: about 0.1 points at the domain level and 0.05 points for the component scores. Associations of this size fall below conventional thresholds for a minimally important difference at the individual level, and larger gains would require larger increases in activity. Differences between individuals were more substantial: participants whose average activity differed by the interquartile range differed in BMI by approximately 1.7 units, although such between-person comparisons are more susceptible to confounding. The clinical relevance of these findings therefore rests less on the size of any single association than on the consistency of the direct pathway across nearly all HRQoL domains and on the potential population-level impact of modest shifts achieved at scale. Interestingly, among the three components of physical activity (frequency, intensity, and single-session duration), we only observed this association for frequency, which remained the case when the three components were analysed simultaneously, in which the between-person association with intensity was also no longer evident. This pattern is notable given that much of the randomized trial literature emphasizes higher-intensity training [30], whereas in real-world populations with excess weight and common comorbidities, increasing the frequency of lower-intensity activity may be both more feasible and more impactful. Extending activity frequency may also be easier to modify behaviourally than, for example, increasing intensity, and may therefore represent a key pathway through which lifestyle interventions can support sustainable improvements in physical activity and downstream weight-related outcomes.

Evidence regarding the effectiveness of digital interventions in promoting physical activity has been mixed: some meta-analyses report no significant pooled effect, likely reflecting methodological heterogeneity across intervention formats and measurement methods [11], whereas others targeting individuals with overweight or obesity indicate small-to-moderate improvements in physical activity [12]. Importantly, digital weight loss interventions that actively promote physical activity have demonstrated their potential to produce significant reductions in BMI, waist circumference, and body fat percentage [31]. Consistent with this, in the current analyses higher physical activity was systematically associated with lower BMI across all models, supporting its role as an important behavioural pathway linking lifestyle change to downstream health benefits. In terms of weight loss, comprehensive digital interventions that combine physical activity with additional supportive resources, such as those in the HWC program, may offer enhanced benefits compared to approaches focused solely on physical activity.

Response to the follow-up questionnaires declined markedly over the program. High attrition is characteristic of digital health interventions rather than specific to the HWC program [32], but it limits what can be concluded about effectiveness as delivered: the present estimates describe associations among those who continued to report, not the average benefit across all who enrolled. In the same program, discontinuation over 12 months has previously been reported at 29.1% [16], considerably lower than the proportion without HRQoL data here, indicating that the missing follow-up largely reflects non-response to the questionnaires rather than withdrawal from the intervention. Improving response and adherence, through shorter and less frequent assessments, automated reminders, and contacts with coaches, is nonetheless a priority both for real-world digital programs and for the interpretation of their outcomes [33].

A major strength of the present study is its basis in a large, nationwide real-world digital lifestyle intervention, an evidence base that remains extremely limited compared with traditional weight loss trials. Unlike conventional randomized controlled trials, our study includes a broad and clinically heterogeneous population that more closely reflects the patients encountered in routine obesity care. An additional strength is the use of a longitudinal mediation framework to disentangle the relative contributions of physical activity and weight loss to changes in HRQoL, an approach that has been rarely applied in real-world lifestyle interventions. Modelling physical activity, BMI, and HRQoL as time-varying processes assessed at identical occasions allowed us to examine whether within-person variations in BMI mediated the association.

Among the limitations, the underrepresentation of men, while common across weight-loss interventions, may limit the direct extrapolation of the findings. However, given the physiological and behavioural mechanisms linking increased physical activity to improvements in weight and HRQoL, there is little reason to expect that the core associations observed here would not apply more broadly across sexes. Nonetheless, improving engagement strategies for men remains an important area for future work. As is common in large-scale digital interventions, most data were self-reported. Self-reported weight may introduce some degree of underestimation; however, the anonymity afforded by the digital format may reduce social desirability bias, and the wide distribution of reported weights suggests that systematic underreporting is unlikely to meaningfully bias the observed associations. HRQoL and physical activity were also reported subjectively, but these measures capture the lived-experience outcomes that lifestyle interventions aim to influence, and the consistency of patterns across domains supports their validity in this context. Completion of the RAND-36 declined over time, with follow-up HRQoL data available for 19% of participants at 12 months, although non-response does not indicate discontinued from the program. Those with follow-up data were older, more often women, and had lower BMI, but differed only modestly in baseline HRQoL (mainly in mental components). Because these variables are included in the models, a missing-at-random mechanism is plausible, and the within-person estimates on which our conclusions rest are less sensitive to between-person selection. Data missing not at random cannot, however, be excluded. The observational nature of the study precludes definitive causal inference. While increased physical activity is plausibly an upstream driver of both weight change and HRQoL improvements, reciprocal relationships are also possible. For example, weight loss may increase motivation or capacity for physical activity, or improvements in HRQoL may facilitate further behavioural engagement. The data were collected between 2016 and 2020, and although delivery formats have since evolved, the core behavioural components of the intervention, including promotion of physical activity and weight management, remain central to contemporary digital lifestyle programs. Importantly, the HWC program has undergone iterative updates to reflect advances in evidence and clinical practice, suggesting that the underlying principles remain relevant. Nevertheless, changes in digital delivery formats and user interfaces should be considered when interpreting the external validity of the findings. Importantly, the mechanisms linking physical activity, weight loss, and HRQoL are unlikely to be substantially altered by changes in digital delivery platforms.

## Conclusion

Participation in the HWC program, a 12-month multimodal online lifestyle intervention, was associated with an increase in reported physical activity. Follow-up questionnaire data were, however, available for a minority of participants at 12 months. Periods of higher-than-usual physical activity were consistently associated with lower BMI and with better HRQoL. The association with HRQoL was largely independent of BMI and was evident for both the physical and mental components. BMI partially mediated the association with the physical HRQoL domains, but the association between physical activity and HRQoL was predominantly direct. The magnitude of the individual associations was nevertheless modest. In practice, physical activity merits attention as a treatment goal alongside weight management in digital lifestyle care and supporting sustained engagement is a prerequisite for realising its benefits.

## Supplementary Information

Below is the link to the electronic supplementary material.

Supplementary Material 1

## Data Availability

The data that support the findings of this study are available from Helsinki University Hospital, but restrictions apply to the availability of these data, which were used under license for the current study, and are not publicly available.

## Abbreviations

BMI: Body mass index
HWC: Healthy weight coaching
MET-h/day: Metabolic equivalent of task (MET) hours per day
