# Supplementary material for: Disentangling direct and weight-mediated effects of physical activity on health-related quality of life in a 12-month real-world digital lifestyle intervention
Source: Qual Life Res. 2026 Oct 22;35(11):313. doi: 10.1007/s11136-026-04425-3 (PMC13690927; doi:10.1007/s11136-026-04425-3)
Supplement: Supplementary file 1 [file 11136_2026_4425_MOESM1_ESM.pdf]

Disentangling direct and weight-mediated effects of physical activity on health-related quality of life in a 12-month real-world digital lifestyle intervention

Isabel McCann[1], Anu Joki[2,3], Laura-Unnukka Suojanen[3], Mikko S. Venäläinen[4], Kirsi H. Pietiläinen[2,3]*, Aila J. Ahola[2,3]*

[1] University of South Florida Morsani College of Medicine, Tampa, FL, USA

[2] Obesity Research Unit, Research Program for Clinical and Molecular Metabolism, Faculty of Medicine, University of Helsinki, Helsinki, Finland

[3] Healthy Weight Hub, Abdominal Centre, Helsinki University Hospital and University of Helsinki, Helsinki, Finland

[4] Department of Medical Physics, Turku University Hospital and University of Turku, Turku, Finland

*Corresponding author Kirsi Pietiläinen, Obesity Research Unit, Biomedicum 1, Haartmaninkatu 8, PO Box 63, FI-00014 University of Helsinki, Finland. Tel +358 505992295. Email: kirsi.pietilainen@helsinki.fi

*Corresponding author Aila Ahola, Obesity Research Unit, Biomedicum 1, Haartmaninkatu 8, PO Box 63, FI-00014 University of Helsinki, Helsinki, Finland. Tel +358 505002181, Email: aila.ahola@helsinki.fi

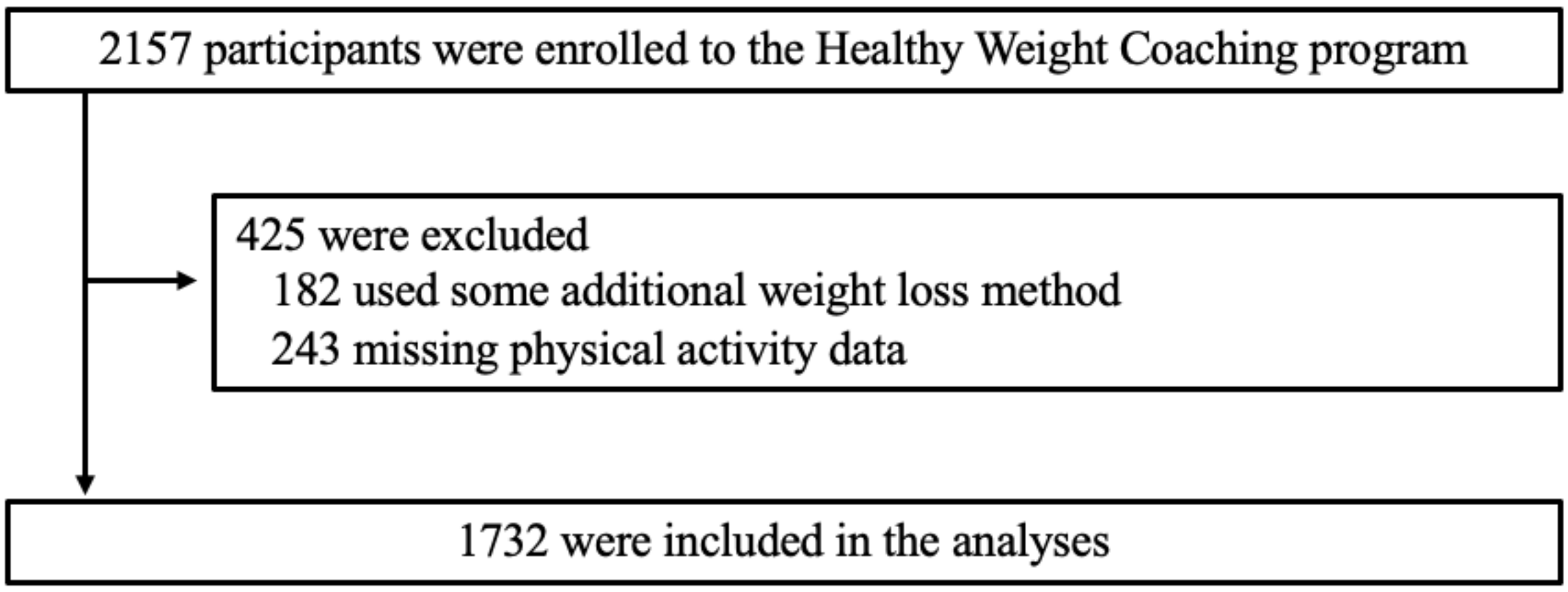

Supplementary Fig 1 Flowchart of participants included and excluded from study population

Supplementary Table 1 Baseline characteristics of individuals who were included and excluded from the study population

	**Included** n=1732 (80.3%)		**Excluded** n=425 (19.7%)		
	N of observations	N (%) or median (IQR)	N of observations	N (%) or median (IQR)	p
Women, %	1732	1436 (82.9)	425	343 (80.7)	0.286
Age, years	1732	52 (42 – 59)	425	53 (42 – 59)	0.526
Weight, kg	1732	110 (98 – 126)	425	114 (99 – 130)	0.012
Body mass index, kg/m^2	1732	39.0 (35.3 – 43.5)	425	40.1 (36.7 – 44.4)	<0.001
MET-h/day	1732	1.5 (0.4 – 3.0)	165	1.1 (0.4 – 2.1)	0.413
HRQoL domains					
Physical functioning	1718	75 (55 – 85)	210	71 (55 – 85)	0.112
Role limitations–physical	1710	50 (25 – 100)	209	50 (0 – 100)	0.562
Bodily pain	1703	58 (35 – 78)	284	58 (45 – 78)	0.626
General health	1715	45 (35 – 60)	210	50 (34 – 65)	0.540
Vitality	1712	55 (40 – 65)	211	55 (45 – 70)	0.073
Social functioning	1675	75 (63 – 100)	206	75 (63 – 100)	0.312
Role limitations–emotional	1674	100 (33 – 100)	206	100 (67 – 100)	<0.001
Mental health	1712	68 (56 – 80)	211	72 (60 – 80)	0.085
Physical component score	1542	41 (35 – 47)	192	40 (34 – 47)	0.266
Mental component score	1542	50 (39 – 57)	192	52 (43 – 58)	0.005

Between-group comparisons were conducted using the Chi-squared test for the categorical variable and the Mann-Whitney U-test for continuous variables. IQR, interquartile range; MET-h/day, metabolic equivalent of task hours per day; HRQoL, health-related quality of life.

Supplementary Table 2 Numbers of observations at each time point

	**Baseline**	**3 months**	**6 months**	**9 months**	**12 months**
Body mass index	1732	1548	1274	1015	558
MET-h/day	1732	1081	726	482	314
Physical functioning	1718		735		312
Role limitations–physical	1710		729		312
Bodily pain	1703		732		317
General health	1715		735		312
Vitality	1712		735		313
Social functioning	1675		709		301
Role limitations–emotional	1674		708		302
Mental health	1712		735		313
Physical component score	1542		655		290
Mental component score	1542		655		290

MET-h/day; metabolic equivalent of task hours/day

Supplementary Table 3 Baseline characteristics of participants with and without health-related quality of life data at the 6- and 12-month time points

	HRQoL data at 6 months			HRQoL data at 12 months		
	Yes, n=757 (43.7%)	No, n=975 (56.3%)	p	Yes, n=329 (19.0%)	No, n=1403 (81.0%)	p
Women, N (%)	658 (86.9)	778 (79.8)	<0.001	291 (88.4)	1145 (81.6)	0.003
Age, years	53 (45 – 60)	50 (39 – 58)	<0.001	55 (46 – 62)	51 (41 – 58)	<0.001
Weight, kg	108 (96 – 124)	113 (99 – 128)	<0.001	107 (95 – 122)	112 (98 – 126)	<0.001
Body mass index, kg/m^2	38.6 (34.9 – 42.8)	39.4 (35.8 – 43.9)	0.003	38.3 (34.4 – 42.5)	39.2 (35.5 – 43.7)	0.009
MET-h/day	1.9 (0.5 – 3.8)	1.1 (0.4 – 2.1)	<0.001	1.9 (0.6 – 3.8)	1.5 (0.4 – 3.0)	0.004
HRQoL domains						
Physical functioning	75 (55 – 85)	75 (55 – 85)	0.595	75 (55 – 85)	75 (55 – 85)	0.429
Role limitations–physical	50 (0 – 100)	50 (25 – 100)	0.703	50 (25 – 100)	50 (25 – 100)	0.812
Bodily pain	58 (45 – 78)	58 (35 – 78)	0.049	58 (45 – 80)	58 (35 – 78)	0.047
General health	50 (35 – 65)	45 (30 – 60)	<0.001	50 (35 – 65)	45 (30 – 60)	<0.001
Vitality	55 (40 – 70)	50 (35 – 65)	<0.001	55 (40 – 70)	50 (40 – 65)	0.005
Social functioning	75 (63 – 100)	75 (50 – 100)	0.011	75 (63 – 100)	75 (50 – 100)	0.011
Role limitations–emotional	100 (33 – 100)	67 (33 – 100)	0.003	100 (33 – 100)	100 (33 – 100)	0.058
Mental health	72 (56 – 80)	68 (52 – 80)	<0.001	72 (60 – 84)	68 (52 – 80)	0.002
Physical component score	41 (35 – 47)	41 (34 – 47)	0.947	41 (35 – 47)	41 (35 – 47)	0.909
Mental component score	51 (41 – 58)	48 (38 – 56)	<0.001	51 (41 – 58)	49 (39 – 56)	0.002

Participants were classified as having follow-up data if at least one of the eight HRQoL domains was available at that time point. Between-group comparisons were conducted using the Chi-squared test for the categorical variable and the Mann-Whitney U-test for continuous variables. HRQoL, health-related quality of life; MET-h/day, metabolic equivalent of task hours per day.

Supplementary Table 4 Full results of the linear mixed models examining longitudinal associations between physical activity and body mass index

Parameter	B (95% confidence interval)	p
Total physical activity (1732 participants, 4209 observations)		
Intercept	47.247 (45.937 to 48.556)	<0.001
MET-h/day – between person	-0.652 (-0.779 to -0.526)	<0.001
MET-h/day – within person	-0.032 (-0.053 to -0.012)	0.002
Sex, men vs. women (reference)	0.658 (-0.127 to 1.443)	0.100
Time		
Baseline (reference)		
3 months	-0.852 (-0.921 to -0.783)	<0.001
6 months	-1.289 (-1.397 to -1.181)	<0.001
9 months	-1.518 (-1.667 to -1.370)	<0.001
12 months	-1.827 (-2.035 to -1.619)	<0.001
Age, years	-0.116 (-0.141 to -0.091)	<0.001
Physical activity – frequency (1732 participants, 4226 observations)		
Intercept	47.366 (46.035 to 48.696)	<0.001
PA frequency – between person	-0.012 (-0.015 to -0.009)	<0.001
PA frequency – within person	-0.001 (-0.001 to -0.001)	0.009
Sex, men vs. women (reference)	0.380 (-0.412 to 1.173)	0.347
Time		
Baseline (reference)		
3 months	-0.851 (-0.921 to -0.782)	<0.001
6 months	-1.291 (-1.398 to -1.183)	<0.001
9 months	-1.519 (-1.667 to -1.371)	<0.001
12 months	-1.835 (-2.043 to -1.628)	<0.001
Age, years	-0.113 (-0.139 to -0.087)	<0.001
Physical activity – single-session duration (1713 participants, 4175 observations)		
Intercept	49.762 (48.372 to 51.152)	<0.001
PA duration – between person	-5.216 (-6.068 to -4.365)	<0.001
PA duration – within person	-0.084 (-0.206 to 0.038)	0.178
Sex, men vs. women (reference)	0.973 (0.189 to 1.757)	0.015
Time		
Baseline (reference)		
3 months	-0.861 (-0.930 to -0.793)	<0.001
6 months	-1.290 (-1.398 to -1.183)	<0.001
9 months	-1.516 (-1.664 to -1.368)	<0.001
12 months	-1.832 (-2.040 to -1.624)	<0.001
Age, years	-0.121 (-0.146 to -0.096)	<0.001
Physical activity – intensity (1722 participants, 4190 observations)		
Intercept	49.111 (47.155 to 51.066)	<0.001
PA intensity – between person	-0.338 (-0.520 to -0.156)	<0.001
PA intensity – within person	0.017 (-0.006 to 0.040)	0.154
Sex, men vs. women (reference)	0.689 (-0.120 to 1.497)	0.095
Time		
Baseline (reference)		
3 months	-0.870 (-0.939 to -0.801)	<0.001

6 months	-1.298 (-1.406 to -1.191)	<0.001
9 months	-1.531 (-1.680 to -1.383)	<0.001
12 months	-1.851 (-2.058 to -1.643)	<0.001
Age, years	-0.138 (-0.164 to -0.112)	<0.001

Body mass index was the dependent variable in all models. Each physical activity variable was analysed in a separate model, with physical activity decomposed into between-person (participant-specific mean across follow-up) and within-person (deviation from the participant-specific mean) components. Reference categories were women for sex and baseline for time. Repeated observations obtained at baseline and at 3, 6, 9, and 12 months were modelled within participants using a first-order autoregressive (AR(1)) covariance structure, estimated by restricted maximum likelihood using all available observations. The intercept corresponds to the model estimate with all covariates set to zero and is not directly interpretable. B, unstandardised regression coefficient; MET-h/day, metabolic equivalent of task hours per day; PA, physical activity.

Supplementary Table 5 Full results of the linear mixed model in which the three components of physical activity were analysed simultaneously in relation to body mass index

Parameter	B (95% confidence interval)	p
Physical activity components analysed simultaneously (1712 participants, 4165 observations)		
Intercept	49.977 (48.091 to 51.863)	<0.001
PA frequency – between person	-0.007 (-0.010 to -0.004)	<0.001
PA frequency – within person	-0.001 (-0.001 to -0.001)	0.020
PA duration – between person	-4.510 (-5.458 to -3.563)	<0.001
PA duration – within person	-0.094 (-0.217 to 0.029)	0.135
PA intensity – between person	-0.017 (-0.203 to 0.169)	0.859
PA intensity – within person	0.021 (-0.003 to 0.045)	0.080
Sex, men vs. women (reference)	0.851 (0.070 to 1.632)	0.033
Time		
Baseline (reference)		
3 months	-0.849 (-0.919 to -0.779)	<0.001
6 months	-1.282 (-1.390 to -1.174)	<0.001
9 months	-1.504 (-1.653 to -1.355)	<0.001
12 months	-1.812 (-2.020 to -1.604)	<0.001
Age, years	-0.112 (-0.137 to -0.086)	<0.001

Body mass index was the dependent variable. Frequency, single-session duration and intensity of physical activity were entered simultaneously into a single model, each decomposed into between-person (participant-specific mean across follow-up) and within-person (deviation from the participant-specific mean) components. Total physical activity (MET-h/day) is the product of these three components and was therefore not included in this model. Reference categories were women for sex and baseline for time. Repeated observations obtained at baseline and at 3, 6, 9, and 12 months were modelled within participants using a first-order autoregressive (AR(1)) covariance structure, estimated by restricted maximum likelihood using all available observations. The intercept corresponds to the model estimate with all covariates set to zero and is not directly interpretable. B, unstandardized regression coefficient; PA, physical activity.
